# MED8/367: Developing an Internet-based Informatics Diploma

**DOI:** 10.2196/jmir.1.suppl1.e51

**Published:** 1999-09-19

**Authors:** DT Parry, D Abernethy, S Cockcroft, A Breton, JD Gillies

**Affiliations:** ^1^University of Otago, DunedinNew Zealand

**Keywords:** Computer Literacy, Graduate Medical Education, Internet

## Abstract

**Introduction:**

We have been running an Internet and CD-ROM based course in Health Informatics in New Zealand, since July 1998. This paper describes the structure of the course, the technology used and the development process and lessons we have learned. Our target audience is working health care professionals, including primary care and hospital doctors, nurses, and managers. The educational goals of the course include skills in:

- Using electronic information sources, databases, presentation and analysis tools, electronic communication and collaboration tools.
- Understanding of the breadth of the field of Health and Medical Informatics (HMI), including basic computing concepts, advantages and disadvantages of the application of IT in HealthCare situations and the concept of professional HealthCare workers as knowledge workers and information users.

**Methods:**

The format of the course is a four semester, part-time course run over the Internet for HealthCare workers throughout New Zealand. Some course material is delivered via CD-ROM; some via the Web and communication takes place over the Internet using synchronous and asynchronous tools. A face-to-face workshop is provided at the start of each semester. At present 45 students are enrolled on the course. Student assessment takes the form of a set of competency tests (one every 3 weeks) covering application of topics covered in the current module. In addition 40% of the student's mark is assigned according to group work (groups are 4-5 students). These projects include a report on a topic of relevance along with web pages and presentations. Marks are also awarded for active participation in the group.

**Results:**

A standard set of questions is provided to each student by the university - the first set of results have been very positive. Feedback forms have been provided and returned at the end of each workshop.

**Conclusion:**

Internet technology has allowed a higher level of communication between staff and students, and student to student than would be possible with conventional means. Off-the-shelf software can support this process and the barriers to this method of teaching can be breached.

